# Supplementary material for: Learning curve and surgical outcome of robotic assisted colorectal surgery with ERAS program
Source: Sci Rep. 2022 Nov 29;12:20566. doi: 10.1038/s41598-022-24665-w (PMC9709162; doi:10.1038/s41598-022-24665-w)
Supplement: Supplementary file 1 — Supplementary Information 1. [file 41598_2022_24665_MOESM1_ESM.zip › LSC Raw data-Ñ[▒K/071/H_ñw╝╨░O▒Kñσ.pdf]

正式摘要(電子簽章：00000188087144)

W55 / W55 ☐ 英文標題(注意:無英文姓名-請補病人基本檔!) ☐【讀取】憑證

臺中榮民總醫院

出院病歷摘要

轉入號：　　轉出號：

[redacted]

**(8)**流水編號	
**(9)**入院日期	109/03/04 (2020) 直腸外科 W55 - 265
**(10)**轉科日期	
**(11)**出院日期	109/03/26 (2020) 住院天數計 22 日

診斷

- 入院：1. Sigmoid colon adenocarcinoma, TTF-1 (focal +), SATB-2 (+), CDX-2(+), and Napsin-A (-), favor primary colon cancer
  2. Lung cancer, RLL, adenocarcinoma, with mediastinal LN and right side malignant pleural effusion, EGFR(+), cT1bN2M1a, stage IVA, s/p pigtail drainage 2018/12/3-12/10. s/p Tarceva since 2018/12/25
  3. COPD under berodual
  4. Chronic Kidney disease, stage III
  5. Type II DM
  6. Hypertension
  7. old stroke
- 出院：1. Sigmoid colon adenocarcinoma, TTF-1 (focal +), SATB-2 (+), CDX-2(+), and Napsin-A (-), favor primary colon cancer, s/p laparoscopic AR on 2020/3/6, pT4aN1cM1c, K-ras mutation(-)
  2. Lung cancer, RLL, adenocarcinoma, with mediastinal LN and right side malignant pleural effusion, EGFR(+), cT1bN2M1a, stage IVA, s/p pigtail drainage 2018/12/3-12/10. s/p Tarceva since 2018/12/25
  3. COPD under berodual
  4. Chronic Kidney disease, stage III
  5. Type II DM
  6. Hypertension
  7. old stroke

主訴

- Bloody stool for at least 3 months

病史

- This 77 years old male has history of
  1. Lung cancer, RLL, adenocarcinoma, with mediastinal LN and right side malignant pleural effusion, EGFR(+), cT1bN2M1a, stage IVA, s/p pigtail drainage 2018/12/3-12/10. s/p Tarceva since 2018/12/25
  2. Sigmoid colon adenocarcinoma, TTF-1 (focal +), SATB-2 (+), CDX-2(+), and Napsin-A (-), favor primary colon cancer
  3. COPD under berodual
  4. Chronic Kidney disease, stage III
  5. Type II DM
  6. Hypertension
  7. old stroke
  He had bloody stool since last Dec., and during hospitalization in CM ward for lung cancer, he had received colonoscopy on 2019/12/25 which revaled sigmoid cancer and pathology proved adenocarcinoma. Abdominal CT on 2020/1/8 revealed wall thicking of Sigmoid colon. Due to old age, family hesitated about operation. However, Hb dropped to 6.9 when he had CM OPD follow up. This time, he was admitted for Hartmanns operation.

體檢發現

- PS ECOG 3
  CXR on 2019/2/11: improved

- Chest CT on 2019/3/28: much improved, PR.
  Chest CT on 2019/6/19: stable, but some new small nodules, nature?
  Chest CT on 2019/9/5: stable lung tumor, but more infiltrates in LLL, in favor of infectious process.
  CXR on 2019/10/3: stable, no obvious change.
  Chest CT on 2019/11/28: stable disease.
  CXR on 2020/2/13: stable disease, no obvious change.
  - General appearance :
    - easy looking , conscious clear , without cardiopulmonary distress
    - Vital signs自動帶入date 體溫(BT) 脈搏(P) 呼吸(R) 血壓(BP) 身高(BH) 體重(BW) BMI
      20200304 35.9℃ 90次/分 18次/分 120/62mmHg 167cm 58.8kg 21.1
    - Vital signs: B.P. 120/62 mmHg , P.R. 90 /min , R.R. 18 /min , B.T. 35.9 ℃
  - HEENT :
    - Eyes: no anemic conjunctiva, no icteric sclera, normal EOM
    - ENT: no otorrhea, no rhinorrhea, no erythematous mucosa, no injected tonsil, no deformity
  - Neck: supple, freely movable, trachea midline,no deformity, no engorged jugular vein , no palpable thyroid or lymph node enlargement
  - Chest:
    - Inspection: Grossly normal, symmetric and fully expansion, no accessory muscle used, no operation scar, no spider angiomata, no gynecomastia
    - Palpation: no palpable mass or nodule, no subcutaneous emphysema, no tenderness
    - Ausculation: clear breathing sound
  - Heart:
    - Palpation: no thrill, no heave
    - Percussion: normal heart size
    - Ausculation: regular heart beat, normal S1, S2, no gallop, no murmur
  - Extremities: No deformity, full ranged of motion without limitation, no clubbing finger or nail cyanosis, no pitting edema, no ulcer or pigmentation, no obvious skin lesion, no varicose vein, no bone, joint or muscle tenderness

CRS CONDITION

- Karnofsky performance status: (50) Requires considerable assitance and frequent medical care.
- PE of Abdomen:
- CEA: 3.7 ng/ml
- Imaging Studies:
- Colonoscope report: S-colon cancer
- Sono , CT of abdomen and chest Films:2020/1/8 ABD non-contrast CT
  > Small size of right kidney with thinning of bil. renal cortex, chronic renal disease is considered.
  > Mild ascites.
  > No enlargement of bil. adrenal glands.
  > No obvious enlarged lymphadenopathy can be identified at paraaortic retroperitoneum or pelvis.
  > Atherosclerotic change of thoacic, abdominal aorta and bil. common
  iliac arteries with wall calcifications.
  > Geographic consolidation over the dependent part of RLL of lung, R/O pneumonia. Suggest clinical correlation.
  > Bil. moderate pleural effusion.
  > On NG tube.
  > On foley's catheter with non-distended urinary bladder.
  > DJD change of T-L spine with spur formation.
- Figures:
  1. colon & rectum

手術日期及方法

- 手術日期及方法20200306 1. A 3 x 2 cm ulcerative tumor at middle sigmoid colon, which invaded to the muscular layer but penetrate through the serosa. Enlarged lymph nodes at mesocolon(+), peritoneal seeding (+)
  Borrmann type III, cT4N1M1
  2. No other gross tumor nor polyps was found

- 3. There was no palpable liver metastasis.
  4. Clear ascites: minimal

住院治療經過

- After admission, preopsuvery was done. Heart echo showed moderate MR, LVEF 45%. He received laparoscopic AR on 3/6, he started to try water on 3/8, then liquid diet on 3/10. on 3/11, coffee ground was noted, so NPO was applied and PPI pump was given. After operation, we kept cefazolin+metronidaozle for him. We will keep current treatment for him. He started to try water then NG diet since 3/12. Appetite was good, however, only flatus was noted, no stool passage was noted. KUB showed ileus, so we hold NG diet for him. After his clinical condition improved, he could tolerated NG diet well. Post op diarrhea improved after medication treatment. We removed penrose drain for him. After his clinical condition improved, we arranged MBD and OPD f/u for him.

合併症

一般檢查

- 累積報告：

DATE	TIME	ALB	TP	BIL.T	BIL.D	ALKP	AST	ALT	LDH	CHOL	TG	HDL-C	LDL	UA	NH3	AMY
1090213	1154						15	9								
1090304	1538	3.2		0.29	0.08	97	15	10	143							
1090306	1225	3.2		0.42	0.11		17	11								
1090309	0901	2.8		0.25	0.09		8	4								
1090312	1018	3.4		0.30	0.12		13	3								
1090316	0934	3.1		0.21	0.09		11	3								
1090319	0924	3.0		0.43			14	3								
1090323	0949	3.1		0.4												

DATE	TIME	WBC	RBC	HGB	HCT	MCV	PLT	BLST	NEUT	BAND	SEG	LYM	MON	EOS	BAS
1090213	1154	8930	2.51	6.9	22.6	90.0	338		75.2			12.8	6.2	5.0	0.8
1090304	1531	5950	3.20	8.8	29.1	90.9	314		73.9			14.8	5.9	4.4	1.0
1090306	1217	9220		10.2			283		91.5			4.4	3.0	0.4	0.7
1090309	0859	7460		10.0			236		78.2			9.8	6.6	4.6	0.8
1090312	0953	6180		9.5			239		75.6			11.7	7.3	4.4	1.0
1090316	0926	9090		9.7			268		80.5			8.8	7.0	3.0	0.7
1090319	0901	7840		9.5			286		81.2			7.9	5.6	4.7	0.6
1090323	0912	8270		9.4			338		76.3			10.5	6.5	6.0	0.7

DATE	TIME	NA	K	CL	CA	BUN	CREAT	Mg	P	CRP	ESR	PCT	LIPASE	LACTATE
1090213	1154	142	4.2			35	2.21							
1090304	1538	143	4.4	110	8.2	25	1.88							
1090306	1225	143	3.3			24	1.76							
1090309	0901	143	4.2			11	1.62							
1090312	1018	142	3.8	109	8.2	11	1.62							
1090316	0934	138	4.5			20	1.79							
1090319	0924	138	3.8			29	2.21							
1090323	0949	141	4.1			30	1.89							

DATE	TIME	GLUCOSE	PAB	TRF	ZN	CU	KETONE	CK	CKMB	PT-P	PT-C	APTT-P	APTT-C	TRO-T
1090304	1531									10.4	10.1	26.5		
1090304	1538	134												

DATE	TIME	PH	BEB	SCVO2	PO2	HCO3	HGB	PCO2	TCO2
1090316	0728	7.353	-0.5	99.4	164.7	25.2		46.3	26.6

特殊檢查

病理報告

- 活體組織切片病理報告　手術日期：**109/03/06**　病理號：**S0907036**

  診斷醫師:李欣倪 報告日期:109/03/10
  Pathologic diagnosis:
  1. Colon, sigmoid, lapa anterior resection --- Adenocarcinoma, moderately differentiated. Tumor invaded to serosa. The bilateral cut ends and mesocolic lymph nodes (0/16) are free of tumor.
  2. IMA root, dissection --- Unremarkable fibrovascular tissue.
  3. Colon, distal cut end, resection --- Chronic inflammation.
  Ancillary study for diagnosis:

- IHC stains for MLH1, MSH2, PMS2 and MSH6 are done on section E. All above markers are preserved.
  Prognostic and predictive factor:
  1. Tumor size: 2.8x2 cm.
  2. Depth of invasion: Tumor invades to visceral peritoneum (including tumor continuous with serosal surface through area of inflammation).
  3. Histologic grade: G2: Moderately differentiated.
  4. Angiolymphatic invasion: Not identified.
  5. Perineural invasion: Not identified.
  6. Tumor deposits (discontinuous extramural extension): Present, specify number of deposits: one.
  7. Macroscopic tumor perforation: Not identified.
  8. Surgical Margins: a) Proximal Margin: Uninvolved by invasive carcinoma, distance of tumor from margin: 3.5 cm. b) Distal Margin: Uninvolved by invasive carcinoma, distance of tumor from margin: 4.3 cm. c) Radial or Mesenteric Margin: Uninvolved by invasive carcinoma, distance of tumor from margin: 4.5 cm.
  9. Lymph node status: Uninvolved (0/16).
  10. Extranodal involvement: Not identified.
  11. Tumor regression grading S/P CCRT: No known presurgical therapy.
  12. Type of polyp in which invasive carcinoma arose: Not identified.
  13. Additional pathologic findings: Not identified.
  14. Pathological TNM Stage: pT4aN1cM1c (Combined with frozen section S0907002 with peritoneal metastasis)(According to the eighth edition, American Joint Committee on Cancer Staging Guidelines for Tumors).
  15. TNM descriptors: Not applicable.
  Gross description:
  The specimen consists of 1) a segment of colon, 10.5 cm in length and 3.5 cm in proximal cut end circumference, 3.5 cm in distal circumference, and mesocolic soft tissue. A gray tan firm annular elevated and centrally ulcerative tumor, 2.8x2 cm, is noted, 3.5 cm to the nearest cut end. On cross section, the tumor invades to the serosa with 45 mm to the radial margin. The both cut ends are free of tumor grossly. 2) IMA root, a piece of tan yellow soft tissue, 3.3x2.5x1.2 cm. 3) distal cut end, a piece of tan mucosa-covered soft tissue, 2.3x2x0.5 cm. Representative part for section: A) proximal cut end B) distal cut end C-E) tumor F) colon G-H) specimen 2-3 J-L) lymph nodes.
  Note:
  1. The interpretation of MMR panel and recommended studies are as below: (1) No loss of nuclear expression of MMR proteins: low probability of microsatellite instability-high (MSI-H) . (2) Please note: There are exceptions to the above IHC interpretations. These results should not be considered in isolation, and clinical correlation with genetic counseling is recommended to assess the need for germline testing.
  2. This case has been peer reviewed by two doctors.
  #T-59300_2 #M-81403_2 2 1105 S0000F
  #T-C4560_2 #M-09183_2 0 1105 000000

病理報告　手術日期：**109/03/14**　病理號：**R0900036**

診斷醫師:陳志榮 報告日期:109/03/19
Result:
Mutation Not Detected.
Mutation Identified:
N/A.
Interpretation:
Mutation Not Detected in KRAS/NRAS codon 12, 13, 59 and 61.
Specimen Site:
Sigmoid Colon.
Surgical Number:
S0907036E.
Test Description:
The KRAS Mutation Test v2 (LSR) and BRAF/NRAS Mutation Test (LSR) are allele-specific, real-time PCR test for the qualitative detection and identification of exon 2, 3, and 4 mutations in the V-Ki-ras2 Kirsten

- rat sarcoma viral oncogene homolog (KRAS) gene, exon 11 and 15 mutations in the proto-oncogene B-Raf (BRAF) gene and exon 2, 3, and 4 mutations in the neuroblastoma RAS viral oncogene homolog (NRAS) gene from formalin-fixed, paraffin-embedded tissue (FFPET). It is intended for life science research only and is not for use in diagnostic procedures. The mutation can be identified as KRAS G12X (G12A, G12C, G12D, G12R, G12S, G12V), KRAS G13X (G13A, G13C, G13D, G13R, G13S, G13V) , A59X (A59E, A59G, A59S, A59T), KRAS Q61X (Q61E, Q61Hc, Q61Ht, Q61K, Q61L, Q61P, Q61R), KRAS K117X (K117Nc, K117Nt) , KRAS A146X (A146P, A146T, A146V) , BRAF V600E/E2/D (BRAF V600E, V600E2, V600D), BRAF V600K , BRAF V600R , BRAF K601E , BRAF Exon11 (G466A, G466V, G469A, G469R, G469V) , NRAS G12X (G12A, G12C, G12D, G12R, G12S, G12V) , NRAS G13X (G13A, G13C, G13D, G13R, G13S, G13V) , NRAS A18T , NRAS Q61X (Q61Ht, Q61Hc, Q61K, Q61L, Q61P, Q61R) , NRAS Other Ex 3 and 4 (A59D, A59T, K117Nc, K117Nt, A146T.
  Methodology:
  The KRAS Mutation Test v2 (LSR) and BRAF/NRAS Mutation Test (LSR) are based on two major processes: (1) manual sample preparation to obtain genomic DNA from FFPET; and (2) PCR amplification and detection of target DNA using complementary primer pairs and oligonucleotide probes labeled with fluorescent dyes.
  The test is designed to detect 28 unique mutations at a percent mutation of 5% or greater, unless otherwise indicated.
  Mutation detection is achieved through PCR analysis with the cobas z 480 analyzer (Roche Diagnostics Ltd, Switzerland)... A mutant control and negative control are included in each run to confirm the validity of the run.
  Clinical Significance:
  The confirmed KRAS mutation status is critical when evaluating a candidate patient for EGFR-targeted therapy. Mutations in the KRAS gene are associated with poor prognosis and non-response to anti-EGFR therapies. KRAS mutation testing is recommended by the National Comprehensive Cancer Network (NCCN) and the Bureau Of Health Promotion, Department Of Health R.O.C before starting EGFR-targeted therapy in both metastatic colorectal cancer and advanced non-small cell lung cancer.
  Note:
  KRAS mutation test results should be interpreted in conjunction with all other available clinical and laboratory information when evaluating treatment options for cancer patients. Laboratory testing performed by the Department of Pathology of VGHTC. This test was performed with life science research reagents (LSR's). This test was developed and its performance characteristics were determined by the Department of Pathology of VGHTC. As with all LSR's, they have not been cleared or approved by the U.S. Food and Drug Administration, The FDA has determined that such clearance or approval is not necessary.
  #T-59300_2 #M-81403_2 2 1100 000000

其它

出院時情況

- 3.改門診治療

出院指示

藥名	劑量	劑量單位	用法	頻次	數量	數量單位	現況	天數	單位
Xanax tab 0.5mg	1	TAB	PO	BIDPRN	10	TAB	TH	5	天
Smecta powder 3g	1	PKG	PO	TID	15	PKG	TH	5	天
Loperamide cap 2mg	1	CAP	PO	QDPRN	5	CAP	TH	5	天
150mg Tarceva tab	1	TAB	PO	QDAC	14	TAB	TH	14	天
Through tab	1	TAB	PO	QNPRN	5	TAB	TH	5	天
Mag. Oxide tab	2	TAB	PO	TIDPRN	30	TAB	TH	5	天
(□)Rosis tab 40mg	1	TAB	PO	Q2D	5	TAB	TH	5	天
MOSAD tab 5mg	1	TAB	PO	TID	15	TAB	TH	5	天
Flexpen Novorapid	8	U	SC	TIDAC	1	U	TH	5	天
Norvasc tab 5mg	1	TAB	PO	QD	5	TAB	TH	5	天

$Miyarisan BM powd.	1	PACK	PO	TID	15	PACK	TH	5	天
$Biofermin tab	2	TAB	PO	TID	30	TAB	TH	5	天
Nebilet tab 5mg	0.5	TAB	PO	QD	3	TAB	TH	5	天

出院計劃

主治醫師 2062F 蔣鋒帆 / 住院醫師 2303K 陳映菁

正式摘要**(電子簽章：000000188087144)**

W55 / W55

doctorRank:B
